# Erlotinib versus gefitinib for brain metastases in Asian patients with exon 19 EGFR-mutant lung adenocarcinoma: a retrospective, multicenter study

**DOI:** 10.1186/s12890-018-0734-1

**Published:** 2018-11-20

**Authors:** Ye Jiang, Jing Zhang, Juanjuan Huang, Bo Xu, Ning Li, Lei Cao, Mingdong Zhao

**Affiliations:** 1 Department of Neurology, The Affiliated hospital of Hebei University, Yuhua East Road No. 212, Baoding, 071000 Hebei China; 2 Department of Respiratory Medicine, The First Affiliated Hospital, Sun Yat-sen University, No. 58, Zhongshan 2nd Road, Yuexiu District, Guangzhou, 510080 China; 3 Department of Anesthesiology, The Central Hospital of Wuhan, Tongji Medical College, Huazhong University of Science and Technology, Gusao Road No. 16, Jianghan District, Wuhan, 430014 Hubei China; 4 Department of Thoracic surgery, The First Affiliated Hospital, Sun Yat-sen University, No. 58, Zhongshan 2nd Road, Yuexiu District, Guangzhou, 510080 China; 5 Department of Orthopaedics, Jinshan Hospital, Fudan University, Longhang Road No. 1508, Jinshan District, Shanghai City, 201508 China

**Keywords:** Erlotinib, Gefitinib, Lung adenocarcinoma, Overall survival

## Abstract

**Background:**

The purpose of this study was to compare clinical outcomes of Erlotinib versus Gefitinib in the treatment of Asian patients with exon 19 EGFR-mutant lung adenocarcinoma and newly diagnosed brain metastases.

**Methods:**

Consecutive Asian patients with exon 19 EGFR-mutant lung adenocarcinoma and newly diagnosed brain metastases were identified and initially received peroral administration of 150 mg/d erlotinib or 250 mg/d gefitinib during 2009–2015. Overall survival (OS) was the primary endpoint. Progression-free survival (PFS) was the second endpoint.

**Results:**

The cohort consisted of 227 Asian patients (erlotinib-treated cohort: *n* = 112, mean age = 58.5 years [SD: 20.13]; gefitinib-treated cohort: *n* = 115, mean age = 58.4 years [SD: 19.52]). In a multivariate analysis controlling for age, sex and time span of smoking history, significant difference was detected in the 36-month OS between erlotinib and gefitinib groups (58.3% vs. 49.1%, *p* = 0.012). There was also significant difference in the 36-month PFS between erlotinib and gefitinib groups (64% vs. 53%, *p* = 0.013).

**Conclusion:**

For Asian patients with exon 19 EGFR-mutant lung adenocarcinoma and brain metastases, erlotinib was associated with a significantly longer OS and a more prolonged PFS and compared with gefitinib.

## Background

Based on previous studies [1–4], gefitinib or erlotinib, epidermal growth factor receptor mutation - tyrosine kinase inhibitor (EGFR-TKI), has been a successful regimen managing advanced non-small cell lung cancer (NSCLC). Furthermore, the data from randomized controlled trials(RCTs) and other investigations have also indicated that EGFR-TKI has advantageous when used as an initial treatment for Asian patients with EGFR-mutant lung adenocarcinoma and brain metastases [5–7]. Yet overall survival (OS) and progression-free survival (PFS) remain controversial for Asian patients with exon 19 EGFR-mutant lung adenocarcinoma and brain metastases [8–14].

We therefore conducted a retrospective review of Asian patients with exon 19 EGFR-mutant lung adenocarcinoma and brain metastases. To our knowledge, this is the first analysis that directly compares gefitinib against erlotinib as initial treatment for brain metastases following exon 19 EGFR-mutant lung adenocarcinoma. We hypothesized that there would be differences in both OS and PFS between patients treated with gefitinib vs. erlotinib.

## Materials and methods

### Study population and end points

The clinical and molecular characteristics and outcome data for 335 Asian patients with exon 19 EGFR-mutant lung adenocarcinoma and newly diagnosed brain metastases retrieved from a registry database were identified at the 4 medical centres between January 2009 and January 2015. Information regarding erlotinib or gefitinib delivery, disease status and survival was obtained from the medical record. Inclusion criteria: age range: 50~ 70 years; patients harbouring exon 19 EGFR mutation; all patients with stage IV lung adenocarcinoma at initial diagnosis; patients initially receiving peroral administration of 150 mg/d erlotinib or 250 mg/d gefitinib; EGFR mutation testing performed in all patients by the molecular diagnostic core laboratory of the Department of Pathology. Exclusion criteria: patients with de novo EGFR-TKI resistance mutations; previous chemotherapy or radiotherapy; no pre-treatment imaging; discontinuation or interruption of erlotinib or gefitinib; death; refusal; organ failure; severe infectious diseases (e.g., systemic inflammatory response syndrome); mental illness; cognitive dysfunction; uncontrolled diabetes mellitus or hypertension. OS was the primary endpoint. PFS was the second endpoint.

### Definitions of the descriptive variables

OS was defined as the period from treatment initiation to the date of death from any cause. PFS was defined as the period from treatment initiation to the date of disease progression. Lung adenocarcinoma staging was performed according to the 7th edition of the Lung Cancer Stage Classification System [15]. For EGFR mutation testing, tumour specimens from primary lung adenocarcinoma were obtained by either needle biopsy/aspiration prior to EGFR-TKI therapy. Imaging examination was carried out every 2 months to assess the drug-related patient’s response. Lung adenocarcinoma response was assessed in accordance with the Response Evaluation Criteria in Solid Tumours (RECIST) by imaging procedure 1 month after treatment and then every 2 months thereafter or when clinically indicated. Responses to EGFR-TKI were conducted by independent radiological reviews. For OS analyses, patients who were still alive or not lost to follow-up at the primary analysis cut-off date were noted at the final follow-up. Living patients were censored at the date of last contact. DNA which was extracted from formalin-fixed, paraffin-embedded tumour tissue was tested with polymerase chain reaction-based assays, as described by Pan et al. [16].

### Statistical analysis

Categorical variables expressed as the count and percentage were analysed using χ^2^-test or the Mann–Whitney U-test. Continuous numeric variables expressed as the mean and SD were analysed with Student’s t-test. Survival probabilities estimated using the Kaplan-Meier method were compared between groups by the log-rank test. Cox regression analyses were executed to adjust for age, sex and time span of smoking history. Statistical analyses were performed using SPSS (version 24.0; IBM, Inc., Chicago, IL, USA) software. A value of *p* < 0.05 was considered statistically significant.

## Results

### Patient characteristics

**Fig. 1:**
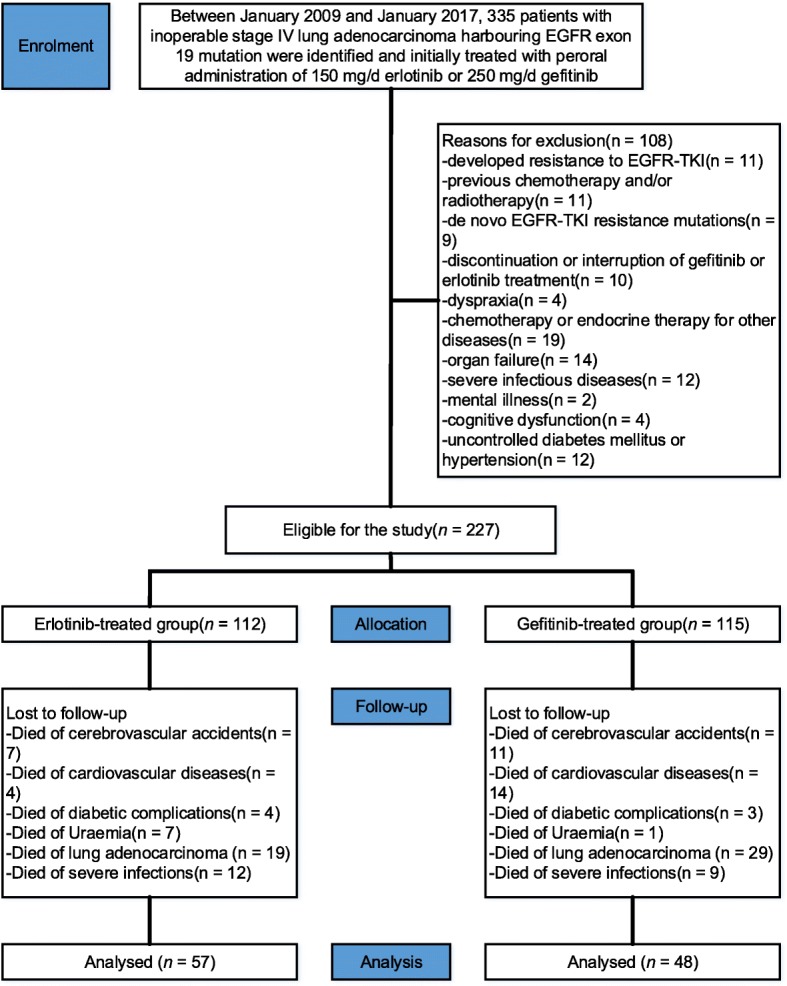
Flow diagram demonstrating the methods to identify the study

In total, 227 Asian patients with exon 19 EGFR-mutant lung adenocarcinoma and brain metastases were included (erlotinib: *n* = 112, mean age = 58.5 years [SD: 20.13]; gefitinib: *n* = 115, mean age = 58.4 years [SD: 19.52]), as summarized in Fig. 1. The comparisons of the demographic characteristics are presented in Table 1. The median follow-up at the primary analysis cut-off date was 36 months (IQR: 14.5–39.6) for the erlotinib group and 36 months (IQR: 13.3–39.2) for the gefitinib group. The time to occurrence of the progression of brain tumours was significantly prolonged after erlotinib compared with gefitinib. No between-group significant differences were detected in regard to drug-related toxicity or intolerable adverse reactions.

**Table 1:** Baseline characteristics between groups

Variable	Erlotinib (*n* = 112)	Gefitinib (*n* = 115)	*p* - value
Age at onset (years)	58.4 ± 19.52	58.5 ± 20.13	0.212^a^
Sex			0.846^b^
Female	85	86	
Male	27	29	
Smoking status			0.644^c^
Never a smoker	67	65	
Former smokers	23	26	
Current smokers	22	24	
Largest size of brain metastasis			0.841^c^
≤ 10 mm	26	28	
> 10 mm	86	87	
Number of brain metastasis			0.764^c^
≤ 3	65	69	
> 3	47	46	
ECOG performance status			0.838^c^
0	33	35	
1	46	43	
2	25	27	
3	8	10	
Neurological symptoms before the initiation of TKIs			0.352^c^
Nausea	6	5	
Headache	3	3	
Depressed level of consciousness	2	2	
Gait disturbance	1	0	
Muscle weakness	0	1	
Dizziness	1	1	
Urinary retention	1	1	
Cognitive disturbance	2	2	
Memory impairment	1	2	
Blurred vision	2	1	

^a^Analysed using independent-samples t-test. ^b^Analysed using chi-squared test. ^c^Analysed using the Mann-Whitney test. *ECOG* Eastern Cooperative Oncology Group. *TKIs* Tyrosine kinase inhibitors

### Survival analysis

Deaths occurred in the erlotinib and gefitinib groups (44.6 and 58.3%, respectively), as presented in Table 2. Twenty-seven cases had recurrences, 9 of which received the conversion from gefitinib to erlotinib, and no significant increase in brain metastases; 5 continued to receive gefitinib, and brain metastases further worsen until they nearly died; 2 terminated the treatment of gefitinib and eventually died; the therapy of 11 cases was unidentified. There were more than 3 metastases (the sites included the brain, bone, lung, liver, and lymph nodes) in 70 patients in the two groups (28 vs. 42 for erlotinib and gefitinib groups, respectively, *p* = 0.06). All tumours detected were histopathologically parallel to lung adenocarcinoma with identical exon 19 EGFR mutation, excluding a second lung tumour as a possibility.

**Table 2:** Survival analysis at final follow-up

Variable	Erlotinib (*n* = 112)	Gefitinib (*n* = 115)	*p* - value
median PFS (months)	10.8(range, 0–21.3)	8.4(range, 0–20.5)	0.014^*a^
median OS (months)	28.3(range, 3.6–36.2)	25.0(range, 3.3–36.3)	0.033^*a^
Deaths, No.	50	67	0.04^*b^
Age(y)	68.1 ± 8.73	67.7 ± 9.34	0.175^c^
Sex			0.133^b^
Female	30	49	
Male	20	18	
Smoking status			0.770^d^
Never a smoker	30	44	
Former smokers	13	10	
Current smokers	7	13	
Largest size of brain metastasis			0.326^d^
≤ 10 mm	11	10	
> 10 mm	39	57	
Number of brain metastasis			0.467^d^
≤ 3	22	25	
> 3	28	42	
ECOG performance status			0.177^d^
0	6	8	
1	15	30	
2	21	22	
3	8	7	

*Statistically significant. ^a^Analysed using the log-rank test; ^b^Analysed using independent-samples t-test; ^c^Analysed using chi-squared test; ^d^Analysed using the Mann-Whitney test. *PFS* progression-free disease-free survival; *OS* overall survival; *ECOG* Eastern Cooperative Oncology Group

**Fig. 2:**
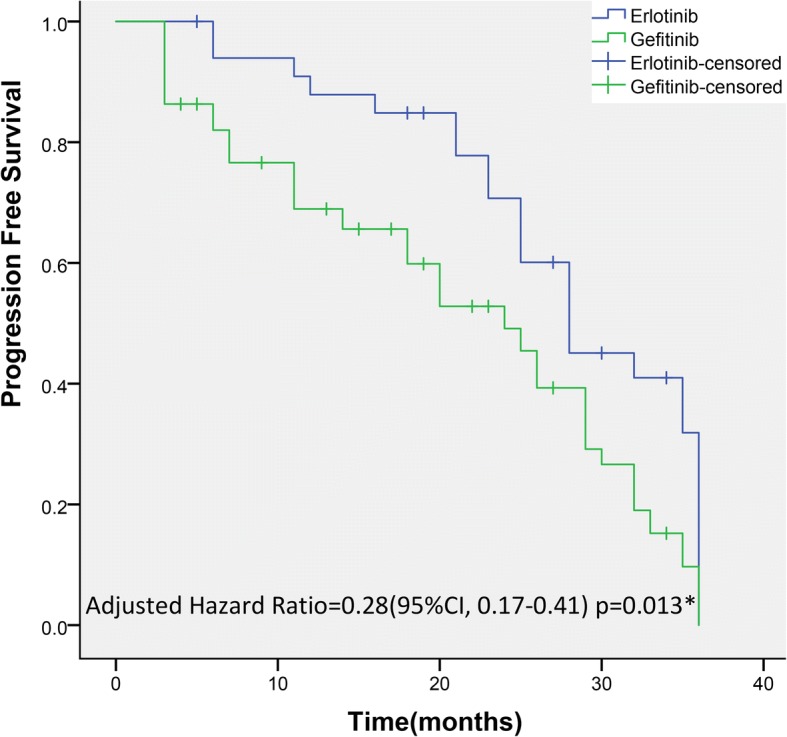
Kaplan–Meier Curves for PFS. The median PFS was 10.8 months (range, 0–21.3 months) in the erlotinib group and 8.4 months (range, 0–20.5 months) in the gefitinib group. A statistically significant difference was detected in PFS between groups. ^*^The hazard ratio was calculated using the Cox proportional hazards model, with age, sex and time span of smoking history as covariates and gefitinib/erlotinib therapy as the time-dependent factor. With respect to PFS, the results were analysed using the log-rank test (*p* = 0.014)

**Fig. 3:**
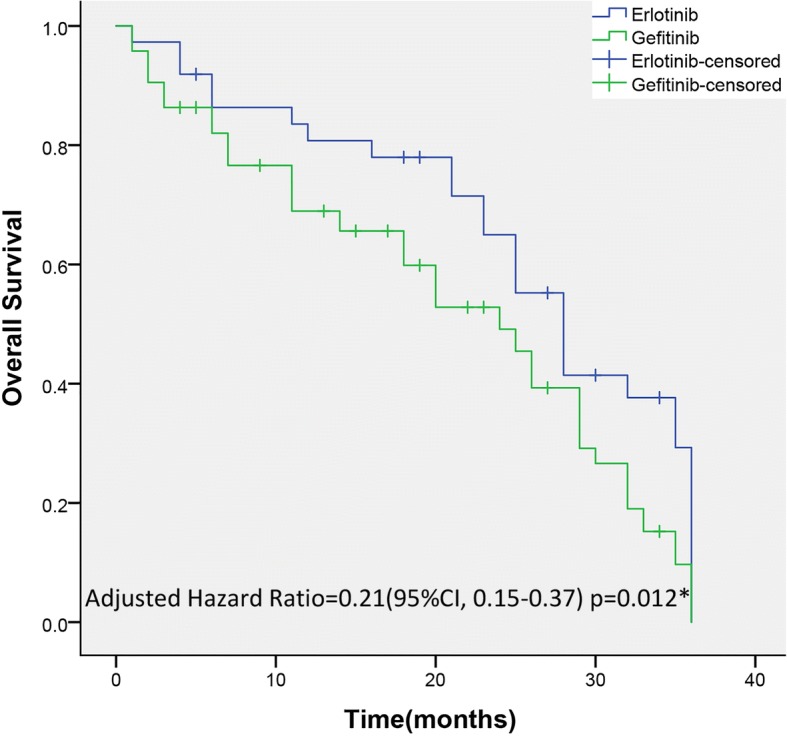
Kaplan–Meier Curves for OS. The median OS was 28.3 months (range, 3.6–36.2 months) in the erlotinib group and 25.0 months (range, 3.3–36.3 months) in the gefitinib group. There was a statistically significant difference in OS between groups. *The hazard ratio was calculated using the Cox proportional hazards model, with age, sex and time span of smoking history as covariates and gefitinib/erlotinib therapy as the time-dependent factor. With respect to the OS, the results were analysed using the log-rank test (*p* = 0.033)

Median PFS and median OS of erlotinib-treated patients were 10.8 months (95% CI: 4 to 16) and 28.3 months (95% CI: 3 to NA), respectively. Median PFS and median OS of gefitinib-treated patients were 8.4 months (95% CI: 4 to 13) and 25.0 months (95% CI: 5 to NA), respectively, as presented in Figs. 2 and 3. A statistically significant difference was detected in median PFS and median OS between groups. Multivariate analysis, after adjusting for age, sex and time span of smoking history, indicated that erlotinib-treated patients had a 36-month PFS rate of 64% compared with 53% for gefitinib-treated patients (HR = 0.28; 95% CI: 0.17–0.41; *p* = 0.013); erlotinib-treated patients had a 36-month OS of 58.3% compared with 49.1% for gefitinib-treated patients (HR: 0.21; 95% CI: 0.15 to 0.37; *p* = 0.012).

## Discussion

In the current study, Asian patients with positive exon 19 EGFR-mutant lung adenocarcinoma and newly diagnosed brain metastases who initially received peroral administration of 150 mg/d erlotinib or 250 mg/d gefitinib were followed for a mean of 36 months, and the most important finding was that erlotinib was associated with a significantly longer OS and more prolonged PFS than gefitinib.

This has increasingly become a consensus that the supreme benefit of EGFR-TKI therapy occurred in patients with EGFR-mutant lung adenocarcinoma and brain metastases [11, 15–19]. The evidence in the previous literature regarding the optimal treatment strategy for the initial management of Asia patients with metastatic EGFR-mutant lung adenocarcinoma was questionable [5, 6, 17], although there are limited randomized trials directing this therapy. To date, there was no solid evidence that gefitinib or erlotinib had less efficacy than afatinib in first-line treatment of patients with EGFR-mutant lung adenocarcinoma and brain metastases [1–4, 12]. Several studies indicated that gefitinib may be superior to erlotinib, but the finding was based on low event numbers and small sample sizes [20–22]. Our findings were in line with previous prospective trials that the response rates to EGFR-TKI therapy in stage IV lung adenocarcinoma patients harbouring exon 19 EGFR mutation ranged from 60 to 70% [13, 22]. Moreover, more studies that compared both OS and PFS between erlotinib and gefitinib in stage IV exon 19 EGFR-mutant lung adenocarcinoma patients after completion of all standard adjuvant chemotherapy and/or radiation therapy also showed similar outcomes [5, 12, 21]. Previous studies established erlotinib was superior to gefitinib in advanced EGFR-mutated patients with leptomeningeal metastases from lung adenocarcinomas that progressed during gefitinib therapy but responded to erlotinib [3, 15, 20, 21].

A retrospective multicenter study by Fan et al. [5] exhibited that median PFS of gefitinib and erlotinib groups was 3.6 and 4.6 months, respectively (*p* < 0.027). Median OS of gefitinib and erlotinib groups was 9.6 and 10.7 months, respectively (*p* < 0.013). Nevertheless, a previous meta-analysis reported by Normando et al. [23] demonstrated no significant difference in the PFS and OS of erlotinib or gefitinib in patients with EGFR-mutant lung adenocarcinoma and brain metastases. Recent studies [20, 24] exhibited that the PFS and OS of gefitinib-treated patients was significantly lower than that of erlotinib-treated patients. In exploratory analysis of EGFR-mutated patients, gefitinib failed to generate a PFS or OS benefit [6, 25]. Considering this was an underpowered study that was terminated early with some cases undergoing a short treatment time, the results did not seem to draw conclusions about the impact of erlotinib or gefitinib. Nevertheless, evidence-based medicine analysis [26] exhibited that the PFS and OS of erlotinib-treated young patients (45–55 years old) failed to be superior to gefitinib-treated young patients. Several studies have reported that gefitinib might be a soothing choice for the initial treatment of patients with EGFR-mutant lung adenocarcinoma and newly diagnosed brain metastases [5, 27, 28]. However, another considered problem is that the results after a failed erlotinib or gefitinib are relatively controversial [4]. Currently, there is no consensus about which drug to use in Asian patients with EGFR-mutant lung adenocarcinoma and brain metastases [29]. In China, 80% of patients prefer receiving gefitinib over erlotinib for brain metastases following EGFR-mutant lung adenocarcinoma. The main reason is that gefitinib has a price advantage, and medical insurance can be reimbursed. Only when gefitinib resistance occur are they willing to accept erlotinib treatment. Thus, further study is compulsory on the effects of familial exon 19 EGFR mutation on Asian ethnicity. Consequently, whether erlotinib is superior to gefitinib in the treatment of young patients with brain metastases following EGFR-mutant lung adenocarcinoma, a prospective randomized controlled study of larger samples is required for clarification. Noteworthy, any data from EGFR-TKI trials that fail to select patients based on molecular and clinical characteristics and EGFR-mutant presence may be misrepresentative.

Erlotinib, a specific EGFR-TKI, has been shown to improve PFS compared with chemotherapy when given as first-line treatment for Asian patients with NSCLC with activating EGFR mutations [12, 30]. A multicentre, open-label, randomised phase 3 trial (EURTAC) [30] which is the first prospective head-to-head phase 3 study has shown that erlotinib had longer PFS and milder side-effects than standard chemotherapy in non-Asian patients with advanced NSCLC and EGFR mutations. A randomised, phase III study(OPTIMAL, CTONG-0802) [31] comparing erlotinib with chemotherapy as first-line treatment of EGFR mutation-positive advanced NSCLC showed erlotinib should be considered standard first-line treatment of patients with advanced NSCLC and EGFR mutations. Our findings were consistent with the OPTIMAL. In our study, some statistical results could not be obtained when comparing the OS and PFS between groups. One potential explanation may be attributed to that the treatment period of some patients was less than 6 months, related to premature death.

As an EGFR-targeted drug for effective treatment of advanced NSCLC, erlotinib’s main drug-related toxicity was rash, mostly mild to moderate [32]. The rashes in most patients in this study were comparable to those in previous studies [11, 31], and the symptoms tended to improve after appropriate treatment. The incidence of grade 3 or 4 adverse events was low. No patient reduced or discontinued treatment due to intolerable adverse reactions.

This study should be interpreted considering important limitations. Firstly, the most important limitation is the retrospective nature, which limits the level of evidence. Many cases were excluded from the analysis owing to lack of baseline data. The excluded cases may introduce bias which is scarcely possible to account for and fails to be representative of the larger sample. Secondly, our findings were also limited by the frequency and length of follow-up. Thirdly, although potential confounders were adjusted by us, other unpredictable factors may also be relevant.

## Conclusion

For Asian patients with EGFR-mutant lung adenocarcinoma and brain metastases, erlotinib was associated with a more prolonged PFS and a significantly longer OS compared with gefitinib. Patients with gefitinib-resistant brain metastases appear to be more suitable for treatment with erlotinib. In addition, if gefitinib or erlotinib were to be assessed again in the adjuvant setting, the proper duration of drug use to maximise efficacy but minimise adverse reaction should not be disregarded. Further follow-up is deserved to verify whether previous findings persist over a longer period.

## Abbreviations

CI: confidence interval
ECOG: Eastern Cooperative Oncology Group
EGFR: epidermal growth factor receptor
HR: hazard ratio
IQR: interquartile range
NSCLC: non-small cell lung cancer
OS: overall survival
PFS: progression-free survival
RCTs: randomized controlled trials
SD: standard deviation
TKIs: tyrosine kinase inhibitors
